# Renoprotective vs. Antihypertensive Dosing of Renin–Angiotensin System Inhibitors in Chronic Kidney Disease: Analysis of Summary of Product Characteristics of Medicinal Products Authorized in Poland

**DOI:** 10.3390/jcm15207771

**Published:** 2026-10-09

**Authors:** Marta Maciejczyk, Anna Maria Dworakowska, Magdalena Bujalska-Zadrożny

**Affiliations:** Department of Pharmacotherapy and Pharmaceutical Care, Faculty of Pharmacy, Medical University of Warsaw, 02-097 Warsaw, Poland

**Keywords:** chronic kidney disease, creatinine clearance, renal dose adjustment, renin–angiotensin system inhibitors, Summary of Product Characteristics

## Abstract

**Introduction:** Renin–angiotensin system inhibitors are central to antihypertensive and nephroprotective treatment in chronic kidney disease. However, renal dosing information in Summaries of Product Characteristics (SmPCs) may differ between products and may not reflect current kidney function assessment. **Objectives:** We systematically evaluated dosing recommendations for single-component ACE inhibitors and angiotensin receptor blockers authorized in Poland, focusing on renal impairment and nephroprotective indications. **Patients and methods:** This cross-sectional content analysis included SmPCs for medicinal products classified under ATC codes C09AA and C09CA. Documents were retrieved from Polish and European regulatory sources. Data were extracted on indications, antihypertensive and nephroprotective dosing, renal dose-adjustment recommendations, renal function parameters, estimation formulas, thresholds, dose limits, and dialysis-related statements. **Results:** The final sample included 282 medicinal products. Hypertension was an approved indication in all products, whereas nephroprotective indications were identified in 106 (37.6%). Creatinine clearance was the predominant renal function parameter for dose adjustment, appearing in 190 products (67.4%). No SmPC used glomerular filtration rate for dose adjustment, although GFR was mentioned in 16 products, mainly in contraindications involving aliskiren. References to renal function estimation formulas were rare; Cockcroft–Gault was explicitly mentioned in a few products, while MDRD and CKD-EPI were absent. Nephroprotective and antihypertensive maximum doses were identical in most products, but differences were identified in 15 (14.2%), mainly lisinopril and benazepril. **Conclusions:** SmPCs for ACE inhibitors and angiotensin receptor blockers authorized in Poland show substantial heterogeneity in renal dosing recommendations. Greater harmonization and alignment with contemporary kidney function assessment would improve clinical applicability.

## 1. Introduction

Cardiovascular diseases (CVD) remain the leading cause of death worldwide [1], with arterial hypertension representing the most prevalent and modifiable cardiovascular risk factor [2]. Hypertension is also a major contributor to the development and progression of chronic kidney disease (CKD), promoting renal injury through increased intraglomerular pressure, albuminuria, and progressive nephron loss [3]. Conversely, declining kidney function further impairs blood pressure regulation, creating a self-perpetuating cycle of renal and vascular damage [4].

In patients with CKD, blood pressure control serves not only to reduce cardiovascular risk but also as a key nephroprotective strategy [5,6]. Lowering blood pressure, particularly systolic blood pressure, reduces intraglomerular hypertension, attenuates albuminuria, and slows the decline in glomerular filtration rate (GFR) [4,7]. Accordingly, the 2024 KDIGO Guideline recommends a target systolic blood pressure below 120 mmHg, if tolerated, in adults with CKD [5]. The 2024 ESC Guidelines recommend a target systolic blood pressure of 120–129 mmHg in most treated patients while emphasizing individualized treatment according to tolerability and comorbidities [3]. More recently, the 2026 ESC Guidelines for cardiovascular disease and chronic kidney disease further highlighted the importance of integrated cardiorenal management and optimization of cardiovascular risk factors, including blood pressure control, in patients with CKD [6].

Although intensive blood pressure reduction may provide additional cardiovascular and renal protection, concerns remain regarding potential adverse effects, including symptomatic hypotension, treatment burden, and acute declines in kidney function, particularly in elderly and multimorbid patients [3,7].

Among currently available therapies, inhibitors of the renin–angiotensin system (RAS), including angiotensin-converting enzyme inhibitors (ACE inhibitors) and angiotensin II receptor blockers (ARBs), remain the cornerstone of treatment in CKD, particularly in patients with albuminuria [5]. Beyond their antihypertensive effects, these agents exert direct nephroprotective actions by preferentially dilating the efferent arteriole, thereby reducing intraglomerular pressure and proteinuria [5,8,9]. The pivotal role of RAS blockade in slowing CKD progression has led KDIGO 2024 to recommend administration of ACE inhibitors or ARBs at the highest approved dose that is tolerated by the patient, as the proven renal benefits in clinical trials were achieved using such dosing strategies [5].

Contemporary CKD management also includes sodium-glucose cotransporter 2 (SGLT2) inhibitors [10,11] and, in selected patients, mineralocorticoid receptor antagonists [5,12], both of which provide additional cardiorenal benefits. Nevertheless, ACE inhibitors and ARBs continue to represent the foundation of pharmacological nephroprotection and the first-line treatment for patients with CKD and albuminuria [5].

While the nephroprotective efficacy of ACE inhibitors and ARBs is well established, their optimal dosing in clinical practice remains challenging [5]. Maximizing nephroprotective benefit frequently requires dose escalation, particularly in patients with progressive CKD, where concerns regarding hyperkalaemia, acute reductions in GFR, and drug tolerability become increasingly relevant [9,12]. Consequently, clinicians must balance the goal of achieving maximal renal protection against safety considerations related to impaired kidney function.

Safe prescribing in CKD is challenged not only by patient-related factors but also by structural inconsistencies between laboratory reporting, clinical guidelines, and regulatory product information [5,13,14,15,16]. In Europe, the Summary of Product Characteristics (SmPC) remains the legally binding reference document for medicinal product use [17,18]; however, renal dosing recommendations are often based on heterogeneous historical data, may rely on different methods of renal function assessment, and are not always aligned with contemporary nephrology guidance [5,13,16,19]. Variability in terminology, thresholds used to define renal impairment, and the methods by which kidney function is expressed may create uncertainty at the point of prescribing and contribute to inconsistent therapeutic decisions [16,19,20,21].

Although ACE inhibitors and ARBs are frequently regarded as class-consistent therapies, individual products differ with respect to pharmacokinetic properties, routes of elimination, regulatory approval history, and the wording of recommendations for patients with renal impairment [19,22]. Consequently, clinicians may encounter divergent advice concerning dose initiation, dose escalation, maximum doses, monitoring requirements, or contraindications despite prescribing agents from the same therapeutic class [16,19,20,21,22].

A systematic evaluation of SmPC content may therefore provide important insights into the consistency, clarity, and clinical applicability of renal dosing recommendations for ACE inhibitors and ARBs [19,22,23]. Such an analysis can help identify discrepancies between products, reveal areas where regulatory information may be ambiguous or incomplete, and support ongoing efforts to improve the alignment of product information with contemporary approaches to kidney function assessment and pharmacotherapy in CKD [5,13,22,23]. This is particularly important in the context of increasing reliance on eGFR-based decision making and ongoing discussion regarding the optimal methods for translating estimates of kidney function into prescribing recommendations [5,13,16,24].

The primary aim of this study was to systematically identify, extract, and organize dosing information contained in the SmPCs for single-component renin–angiotensin system inhibitors belonging to Anatomical Therapeutic Chemical (ATC) Classification System groups C09AA and C09CA, with particular emphasis on recommendations relevant to patients with chronic kidney disease.

Because medicinal products may be authorised through different regulatory pathways within the European Union [18], including centralised, decentralised, mutual-recognition and national procedures, comprehensive analysis of all national SmPC versions across EU Member States would be methodologically challenging and could introduce inconsistencies related to document availability and version control. Therefore, the present analysis was restricted to SmPCs authorised and available in Poland.

## 2. Materials and Methods

### 2.1. Study Design and Data Source

This was a cross-sectional content analysis of SmPCs for medicinal products classified under ATC codes C09AA (ACE inhibitors, plain) and C09CA (angiotensin II receptor blockers, plain) authorized for marketing in Poland. No patient-level data or human participant data were analyzed.

The sampling frame was the comprehensive Polish national medicines registry (the Register of Medicinal Products Authorised for Marketing in the Republic of Poland) a publicly accessible database maintained by the Office for Registration of Medicinal Products, Medical Devices and Biocidal Products [25]. Documents were retrieved on 2 May 2026.

The unit of analysis was a single SmPC corresponding to one medicinal product. Only single-component medicinal products were included. For the primary analysis, all medicinal products belonging to ATC groups C09AA (angiotensin-converting enzyme inhibitors, plain) and C09CA (angiotensin II receptor blockers, plain) were included.

### 2.2. Selection of Medicinal Products

The initial registry filter yielded 222 ACE inhibitor entries (C09AA) and 209 ARB entries (C09CA). Products authorized through the parallel import procedure were excluded to avoid duplicate regulatory documentation. After exclusion, 123 ACE inhibitors and 163 ARB products remained (combined total: 286 entries).

SmPC PDF documents were then obtained from two sources. For products authorized through the centralised EU procedure, documents were retrieved from the European Commission website [26]. For products authorized through the centralised EU procedure, a single SmPC often covered multiple product presentations that were registered as separate entries in the Polish medicines register. The remaining products were retrieved from the Polish e-health platform [27]. One file was identified as a patient information leaflet not SmPC; its data were substituted from a related product sharing the same active substance and authorization.

Four entries were excluded from the analysis set: (i) Aqumeldi (Proveca Pharma Limited, Dublin, Ireland, EU/1/23/1717), which was indicated solely for pediatric heart failure and lacked a hypertension indication, and (ii) three strengths of withdrawn telmisartan product (Telmisartan Teva Pharma, Haarlem, Nederland, EU/1/11/719) whose SmPC was inaccessible. The final study sample comprised 282 entries.

### 2.3. Data Extraction

Data extraction was performed manually using a predefined data extraction form by two independent researchers (A.M.D. and M.M.).

For each registry entry, the following variables were recorded: product name and active substance; ATC code (C09AA or C09CA); authorization procedure type; hypertension indication (yes/no); nephroprotective indication (yes/no) and specific indication category; hypertension dosing (initial, titration, target, maximum); nephroprotection dosing by indication sub-case (where applicable); renal impairment dose-adjustment details (renal function parameter, threshold values, corresponding doses, dialysis notes); and renal function estimation formula mentioned in the SmPC (Cockcroft-Gault, MDRD, CKD-EPI, or none).

### 2.4. Data Analysis

Descriptive statistics were used to summarize the data. Categorical variables are reported as frequencies and percentages. The distribution of renal function parameters was calculated as a proportion of all analyzed products.

#### Ethics Statement

The study was based exclusively on publicly available regulatory documents and did not involve human participants, patient data or biological material. Therefore, ethics committee approval was not required.

### 2.5. Use of Generative Artificial Intelligence Tools

Generative artificial intelligence (GenAI) tools were used to support selected aspects of manuscript preparation and document screening. ChatGPT-5.5 (OpenAI, San Francisco, CA, USA) was used for language and stylistic editing of manuscript drafts. The DeepSeek large language model (DeepSeek AI, Hangzhou, China) was used to assist consistency checks during screening of the included SmPCs. All source documents were reviewed manually by the investigators using a predefined data extraction form. AI-generated outputs were reviewed by the authors and were not used to make final data extraction decisions, perform statistical analyses, interpret the results, or draw scientific conclusions. The authors retained full responsibility for all content of the manuscript.

## 3. Results

Among the 282 analyzed medicinal products, hypertension was listed as an approved indication in all SmPCs. Nephroprotective indications were identified in 106 entries (37.6%). These indications were heterogeneous and could be classified into six categories, most commonly diabetic nephropathy associated with microalbuminuria and proteinuria (42 entries) and diabetic kidney disease (37 entries). Less frequently reported indications included diabetic kidney disease with proteinuria ≥0.5 g/day (17 entries), diabetic nephropathy with microalbuminuria alone (6 entries), diabetic nephropathy in type 1 diabetes with proteinuria (3 entries), and chronic kidney disease without further specification (1 entry) (Table 1).

Products with nephroprotective indications were distributed across all authorization pathways. The largest number of nephroprotection-related SmPCs originated from products authorized through the mutual recognition procedure (34 products), followed by national procedures (28 products), centralized procedures (24 products), and decentralized procedures (20 products) (Table 2).

### 3.1. Renal Function Parameter Distribution

The renal function parameter used for dose-adjustment recommendations varied across SmPCs (Table 3). Creatinine clearance was the most commonly used parameter, appearing in 190 products (67.4%). Forty-three products (15.2%) referred to renal impairment without specifying a quantitative renal function parameter, whereas 49 products (17.4%) stated that no dose adjustment was required. Glomerular filtration rate was never used for the purpose of dose adjustment and mentioned only in 16 products (5.6%) in other contexts, mainly with reference to contraindication for use in combination with aliskiren.

### 3.2. Renal Function Estimation Formula Mention

References to specific renal function estimation formulas were rare. Three products (1.0%) explicitly instructed the use of the Cockcroft–Gault equation, while five products (1.8%) provided the equation within the SmPC text. No SmPC mentioned MDRD or CKD-EPI equations. Furthermore, none of the reviewed documents provided specific instructions regarding body-weight adjustment when applying renal function estimates.

### 3.3. Renal Dose-Adjustment Threshold Patterns by Active Substance

Renal dose-adjustment recommendations differed substantially between active substances (Table 4). Most products provided renal dosing guidance based on creatinine clearance thresholds, although the complexity of recommendations varied markedly. Some active substances, such as captopril, perindopril, ramipril, and lisinopril, included detailed dose-adjustment schemes with multiple renal function categories and corresponding dose limits. In contrast, several ARBs, including losartan, irbesartan, and azilsartan, generally stated that no dose adjustment was required in renal impairment, although additional precautions or lower initial doses were sometimes recommended for patients receiving haemodialysis.

For most active substances, renal dosing recommendations were consistent across products containing the same active substance. An exception was quinapril, for which two distinct dose-adjustment schemes were identified despite the presence of the same active ingredient. The observed recommendations ranged from detailed creatinine-clearance-based algorithms to qualitative statements indicating limited clinical experience in severe renal impairment.

### 3.4. Nephroprotective Dosing Recommendations

All 42 ramipril-containing products included in the analysis had identical nephroprotection dosing recommendations across three clinical sub-cases, as specified in their SmPCs (Table 5).

Among the 106 entries listing nephroprotective indications, nephroprotection-specific dosing information was provided in all SmPCs. In the majority of products, the maximum dose recommended for nephroprotective use was identical to the maximum dose approved for hypertension treatment. Overall, 88 products (83.0%) shared the same maximum dose for both indications.

Differences between antihypertensive and nephroprotective dosing recommendations were identified in 15 products (14.2%). These differences were observed mainly among lisinopril-containing products, for which nephroprotective dosing was generally limited to 20 mg/day despite antihypertensive maximum doses ranging from 40 to 80 mg/day. A similar pattern was observed for benazepril-containing products.

In addition to differences in maximum doses, several SmPCs included indication-specific nephroprotective titration schemes. This was particularly evident for ramipril products, which provided separate dosing algorithms for diabetic nephropathy with microalbuminuria, diabetic patients with cardiovascular risk factors, and non-diabetic proteinuric nephropathy. By contrast, losartan and irbesartan products generally used nephroprotective target doses corresponding to their maximum approved antihypertensive doses (100 mg/day and 300 mg/day, respectively), although dedicated nephroprotective dosing descriptions remained available within the SmPCs.

## 4. Discussion

The present study demonstrates substantial heterogeneity in renal dosing recommendations provided in SmPCs for ACE inhibitors and ARBs authorized in Poland. Although creatinine clearance was the predominant parameter used for dose adjustment, considerable variability was observed in the structure, detail, and presentation of renal dosing recommendations. While some SmPCs provided detailed creatinine-clearance-based dosing algorithms, others relied on qualitative descriptions of renal impairment or stated that no dose adjustment was required. In addition, nephroprotective indications and dosing recommendations were not uniformly represented across products containing RAS inhibitors.

A key finding of our analysis was the predominance of creatinine clearance as the renal function parameter used for dose adjustment. This is consistent with the historical development of many renal dosing recommendations, which were often based on pharmacokinetic studies using creatinine clearance rather than contemporary eGFR equations [13,28]. However, this approach creates practical challenges in current clinical practice, where kidney function is most commonly reported as eGFR calculated using CKD-EPI or, less frequently, MDRD equations [29]. This issue has gained additional relevance following the publication of the 2026 ESC Guidelines on cardiovascular disease and chronic kidney disease, which emphasize systematic CKD identification, risk stratification, and integration of contemporary kidney function assessment into cardiovascular care [6]. The continued reliance on creatinine-clearance-based dosing recommendations in SmPCs may therefore create an increasing disconnect between regulatory documents and current multidisciplinary CKD management. Notably, none of the analyzed SmPCs used GFR or eGFR as the renal function parameter for dose adjustment. Although GFR thresholds were occasionally mentioned, these references were related primarily to contraindications or warnings, particularly concerning concomitant use with aliskiren, rather than to dosing recommendations. Explicit references to modern eGFR-based estimation methods such as CKD-EPI or MDRD were entirely absent. Importantly, the significance of this observation extends beyond Poland. Most analyzed medicinal products were authorized through European regulatory procedures, including centralized, decentralized and mutual-recognition pathways. Consequently, the predominance of creatinine-clearance-based recommendations observed in this study likely reflects broader regulatory and historical approaches to renal dosing within the EU [13,19,21] rather than a phenomenon unique to Poland. As laboratory reporting throughout Europe increasingly relies on CKD-EPI-based estimated GFR, the mismatch between routinely reported kidney function measures and the parameters used in product information may affect prescribing decisions across multiple healthcare systems [5,14,29]. Therefore, these findings may be relevant to clinicians, pharmacists, and regulators throughout Europe.

The clinical relevance of this issue is particularly important in CKD, where dose individualization is central to safe pharmacotherapy. Patients with impaired kidney function are at increased risk of drug accumulation, adverse drug reactions, and treatment discontinuation [28,30]. For RAS inhibitors, dose optimization is further complicated by the need to balance nephroprotective efficacy against potential risks, including hyperkalaemia, acute declines in GFR, and hypotension [5,9]. Current CKD management guidelines emphasize the use of ACE inhibitors or ARBs at the highest approved and tolerated dose in patients with albuminuric CKD, because renal benefits in clinical trials were achieved using such dosing strategies [5]. At the same time, SmPCs may impose renal-function-dependent restrictions or provide insufficiently specific guidance, potentially complicating the implementation of guideline-directed nephroprotective therapy.

Our findings also show that antihypertensive and nephroprotective dosing should not be treated as interchangeable. Although nephroprotective dosing information was provided separately, the maximum nephroprotective dose was identical to the maximum antihypertensive dose in most products (83.0%). Differences were primarily observed for selected active substances such as lisinopril and benazepril. For some active substances, nephroprotective indications were associated with specific titration schemes or target doses that differed from the maximum doses used for hypertension treatment. This distinction is clinically relevant because the dose required to achieve blood pressure control may not necessarily correspond to the dose associated with optimal albuminuria reduction or renal protection [3,5]. Conversely, in patients with advanced CKD, renal safety considerations may limit dose escalation even when guideline recommendations support maximization of RAS blockade [5,28]. These observations support the need for SmPCs to distinguish more clearly between antihypertensive and nephroprotective dosing objectives.

The variability observed in our study is consistent with previous reports indicating that renal dosing information may differ substantially between drug information sources and that such discrepancies can contribute to inappropriate prescribing in patients with CKD [16,20,31]. Earlier studies have emphasized that inconsistencies in renal dose adjustment are not merely a technical problem, but a clinically relevant barrier to safe prescribing [16,20,30,31]. Similar concerns have been raised regarding the quality, transparency, and usability of information contained in SmPCs [23].

Our analysis extends these observations to a clinically important group of drugs used both for hypertension control and renal protection, showing that even within a well-established pharmacological class, regulatory recommendations may remain heterogeneous and difficult to apply consistently. The identified discrepancies were observed not only between active substances but also, in selected cases, between products containing the same active ingredient, as demonstrated by the distinct quinapril dose-adjustment schemes identified in the analyzed SmPCs.

The European regulatory framework provides general principles for describing dosing in patients with renal impairment. The European Medicines Agency (EMA) guideline on pharmacokinetic evaluation in renal impairment recommends that dosing instructions should be linked as precisely as possible to renal function measures used in clinical studies and, where appropriate, should include clear dose-adjustment thresholds [13]. The European Commission guideline on SmPCs also indicates that dose recommendations for special populations, including patients with renal impairment, should be clearly presented in the relevant sections of the product information [32]. Nevertheless, our findings suggest that the implementation of these principles varies across individual products. In particular, many SmPCs did not specify the renal function equation, whether renal function should be expressed as absolute or body-surface-area-normalized values, or which patient characteristics should be considered when applying dose recommendations. This may be especially problematic in older patients, individuals with extreme body weight, and patients with advanced CKD, in whom different renal function estimates may lead to different dosing decisions [28,29].

From a clinical perspective, greater harmonization of renal dosing recommendations would be beneficial. Standardized presentation of renal dose adjustment, including the renal function parameter, the estimation method, numerical thresholds, starting doses, maximum doses, and dialysis-related recommendations, could improve the usability of SmPCs in routine practice [13,17,18]. Such harmonization would not necessarily require identical dosing recommendations across products, because pharmacokinetic properties differ between substances. However, it would require a more consistent structure and terminology, enabling clinicians to identify relevant dosing information quickly and apply it safely. Beyond its clinical implications, harmonization may also support regulatory consistency across the European Union. Standardized terminology, renal-function metrics, and dose-adjustment formats could improve the transferability of prescribing guidance across healthcare systems and facilitate implementation of guideline-directed therapy.

This issue is particularly relevant because CKD management recommendations continue to evolve. The KDIGO 2024 guideline reinforces the importance of RAS inhibitors in albuminuric CKD and recommends their use at the highest approved dose that is tolerated [5]. In parallel, the 2024 ESC guidelines emphasize intensive but individualized blood pressure control, taking into account tolerability and comorbidities [3]. These clinical recommendations increase the importance of clear regulatory information, because prescribers must reconcile the goal of maximizing nephroprotection with the need to avoid renal and electrolyte-related adverse events. Periodic review and updating of SmPCs may therefore be necessary to maintain alignment between regulatory documents, contemporary clinical guidelines, and current prescribing practice.

The main strength of this study is its focused evaluation of SmPCs for ACE inhibitors and ARBs, two drug classes with well-established antihypertensive and renoprotective roles in CKD. By analyzing regulatory documents rather than secondary drug information sources, this study addresses the primary source of approved prescribing information used within the European regulatory framework. Another strength is the clinically oriented approach, which considered not only the presence of renal dose-adjustment recommendations but also their practical applicability, including renal function thresholds, dose limits, and the distinction between antihypertensive and nephroprotective dosing.

Several limitations should be acknowledged. First, the analysis was restricted to SmPCs authorized and available in Poland. Although these documents include products authorized through different regulatory pathways, the findings cannot be assumed to represent all national SmPC versions across the European Union. Second, the study evaluated regulatory documents rather than real-world prescribing behavior; therefore, it cannot determine whether the observed heterogeneity directly affects clinical decisions or patient outcomes. Third, the analysis was limited to ACE inhibitors and ARBs and did not include other drug classes with nephroprotective effects, such as SGLT2 inhibitors or mineralocorticoid receptor antagonists. Finally, SmPCs are regulatory documents and may not fully capture the complexity of individualized treatment decisions in patients with CKD, particularly in multimorbid patients or those receiving combination therapy.

Future studies should evaluate whether variability in SmPC-based renal dosing recommendations influences prescribing decisions among physicians and pharmacists. Further research should compare dose-adjustment strategies used in clinical trials, regulatory documents, clinical guidelines, and real-world practice to identify at which stage discrepancies arise, whether during evidence generation, regulatory translation, document updating, or clinical implementation.

More importantly, the heterogeneity identified in the present study may reflect broader limitations of the current evidence base. Although ACE inhibitors and ARBs are recommended as cornerstone therapies for albuminuric CKD, evidence informing optimal renoprotective dosing strategies across different CKD etiologies, CKD stages, age groups, and clinical settings remains limited [5,9,24]. As a result, regulatory recommendations may not always provide sufficiently detailed guidance for the diversity of patients encountered in routine practice.

Large-scale observational studies and multinational registries could help address these evidence gaps by evaluating real-world dosing patterns and their association with renal and cardiovascular outcomes. Such studies should include patients with diabetic and non-diabetic CKD, incorporate contemporary eGFR-based kidney function assessment, and account for age, comorbidities, and concomitant therapies. The present study may therefore serve as a starting point for future multinational research aimed at strengthening the evidence base underlying regulatory dosing recommendations and improving alignment between product information, clinical guidelines, and everyday clinical practice.

## 5. Conclusions

This study demonstrates substantial heterogeneity in renal dosing recommendations provided in SmPCs for ACE inhibitors and ARBs authorized in Poland. Although these medicines play a central role in both antihypertensive and nephroprotective treatment of patients with CKD, SmPCs differed considerably in the structure, terminology, and precision of renal dose-adjustment information. Creatinine clearance remained the predominant renal function parameter, whereas explicit recommendations on contemporary eGFR-based approaches were largely absent. The findings highlight the need for greater harmonization and regular updating of renal dosing information in SmPCs to improve consistency, transparency, and clinical applicability. Given the shared regulatory framework within the European Union, these observations may also be relevant to other European healthcare systems. They support broader efforts to align regulatory product information with contemporary approaches to kidney function assessment. Clearer alignment between regulatory documents, contemporary CKD guidelines, and clinical practice may support safer and more effective use of RAS inhibitors in patients with impaired kidney function.

## Figures and Tables

**Table 1:** Nephroprotection indication categories among products listing nephroprotection as an approved indication (*n* = 106).

Indication Category	Entries, *n*	Key Active Substances
Diabetic nephropathy with microalbuminuria and proteinuria	42	Ramipril
Diabetic kidney disease	37	Irbesartan, lisinopril
Diabetic kidney disease with proteinuria (≥ 0.5 g/day)	17	Losartan
Diabetic nephropathy with microalbuminuria	6	Lisinopril
Diabetic nephropathy (type 1 DM) with proteinuria	3	Captopril
Chronic kidney disease	1	Benazepril

DM, diabetes mellitus.

**Table 2:** Distribution of hypertension and nephroprotection indications by authorization procedure type (*n* = 282).

Procedure Type	Hypertension, *n* *	Nephroprotection, *n* †
CEN	41	24
DCP	98	20
MRP	74	34
NAR	69	28
Total	282	106

* Total entries analysed (*n* = 282), † Entries with nephroprotective indications (*n* = 106).

**Table 3:** Distribution of renal function parameters used for dose-adjustment recommendations in the analyzed entries (*n* = 282).

Renal Function Parameter	Entries, *n* (%)
Creatinine clearance (CrCl)	190 (67.4)
Glomerular filtration rate (GFR)	0 (0)
Not specified (referred, i.e., severe kidney disease, hemodialysis)	43 (15.2)
No dose adjustment required	49 (17.4)

**Table 4:** Key renal impairment dose-adjustment threshold patterns by active substance among analyzed SmPCs (*n* = 282).

Active Substance	Entries, *n*	Renal Function Parameter	Threshold Pattern
Telmisartan	45	—	Mild–moderate RI: no adjustment (*n* = 45); Severe RI/HD: initial 20 mg (*n* = 43)
Ramipril	42	CrCl	≥60 mL/min: no change (max 10 mg); 30–60: no change (max 5 mg); 10–30: initial 1.25 mg (max 5 mg); HD: 1.25 mg post-dialysis
Valsartan	36	CrCl	>10 mL/min: no adjustment; <10: no data available
Irbesartan	26	—	No dose adjustment required (HD: consider lower initial 75 mg)
Candesartan	18	CrCl	<15 mL/min: use with caution; HD and general RI: initial 4 mg, titrate individually
Losartan	17	—	No dose adjustment required (HD: no separate dosing)
Perindopril	17	CrCl	≥60: 4–5 mg; 30–60: 2–2.5 mg; 15–30: 2–2.5 mg every other day; <15/HD: 2–2.5 mg on dialysis days
Lisinopril	17	CrCl	31–80: 5–10 mg; 10–30: 2.5–5 mg; <10/HD: 2.5 mg; max 40 mg
Olmesartan	15	CrCl	20–60: max 20 mg; <20: not recommended
Cilazapril	11	CrCl	>40: standard dose; 10–40: reduced dose; <10: not recommended
Enalapril	9	CrCl	Thresholds at <80, <30, and <10 mL/min
Quinapril	9	CrCl	Two patterns: (i) >60: 10 mg; 30–60: 5 mg; 10–30: 2.5 mg; <10: no data (*n* = 5); (ii) <40: initial 2.5 mg (*n* = 4)
Captopril	6	CrCl	>40: 25–50 mg (max 150); 21–40: 25 mg (max 100); 10–20: 12.5 mg (max 75); <10: 6.25 mg (max 37.5)
Trandolapril	3	CrCl	30–70: standard dose; < 30: initial 0.5 mg
Imidapril	3	CrCl	30–80: 2.5 mg; 10–29: not recommended; <10: contraindicated
Benazepril	3	CrCl	≥30: standard dose; <30: initial 5 mg (max 10 mg)
Azilsartan	3	—	No dose adjustment required
Zofenopril	2	CrCl	<45: half dose; HD: quarter dose

CrCl, creatinine clearance; HD, haemodialysis; RI, renal impairment; SmPC, Summary of Product Characteristics.

**Table 5:** Nephroprotection dosing recommendations for ramipril products (*n* = 42), as stated in Section 4.2 of the SmPC.

Clinical Sub-Case	Initial Dose	Titration Schedule	Target Dose	Maximum Dose
DM with microalbuminuria	1.25 mg	1.25 → 2.5 mg (2 weeks) → 5 mg (2 weeks)	5 mg	5 mg
DM with cardiovascular risk factors	2.5 mg	2.5 → 5 mg (1–2 weeks) → 10 mg (2–3 weeks)	10 mg	10 mg
Non-DM nephropathy (proteinuria ≥ 3 g/day)	1.25 mg	1.25 → 2.5 mg (2 weeks) → 5 mg (2 weeks)	5 mg	5 mg

DM, diabetes mellitus.

## Data Availability

All data analyzed in this study were obtained from publicly available regulatory documents cited in the manuscript.
